# Impact of coronary angiography early after CABG for suspected postoperative myocardial ischemia

**DOI:** 10.1186/s13019-019-0876-0

**Published:** 2019-03-12

**Authors:** Leopold Rupprecht, Christof Schmid, Kurt Debl, Dirk Lunz, Bernhard Flörchinger, Andreas Keyser

**Affiliations:** 1 Department of Cardiothoracic Surgery, University Medical Center Regensburg, Franz-Josef-Strauss-Allee 11, 93053 Regensburg, Germany; 2 Department of Internal Medicine II/Cardiology, University Medical Center Regensburg, Franz-Josef-Strauss-Allee 11, 93053 Regensburg, Germany; 3 Department of Anesthesiology, University Medical Center Regensburg, Franz-Josef-Strauss-Allee 11, 93053 Regensburg, Germany

**Keywords:** Coronary bypass surgery (CABG), Myocardial infarction, Coronary angiography

## Abstract

**Background:**

The incidence of perioperative myocardial infarction is reported to 2–8%. The aim of the study (retrospectively registered) was to evaluate whether control coronary angiography after surgery is useful in case of suspected postoperative myocardial ischemia.

**Methods:**

All patients who demonstrated signs of myocardial ischemia post CABG and underwent coronary angiography from 6/2008 to 06/2015 were retrospectively analyzed. Myocardial ischemia post CABG was defined as an increase of CK/CK-MB, occasionally associated with arrhythmias or low output syndrome.

**Results:**

Overall, 108 patients (age 66 ± 9 years) demonstrated signs of myocardial ischemia post CABG and underwent coronary angiography corresponding to an incidence of 2.2%. Of them, 70 patients (65%) demonstrated graft pathologies. A therapeutic consequence was drawn in 62 Patients (57%), which consisted of redo surgery in 10 patients (9%) and PCI with stent placement in 52 patients (48%). Of the remaining 46 patients, 29 patients showed intact bypass grafts (27%), whereas 17 patients had minor pathologies (16%). Demographic data including the extent of the coronary artery disease, urgency of operation, comorbidities, EuroScore, surgical technique, postoperative lab tests and transfusion requirements were comparable among the groups. Redo surgery patients had prior PCI in 33% of patients, which was much higher than in the other groups. Patients with reintervention had a 30d-mortality rate of 13%, conservatively treated patients only 2.2%. Mortality was highest after redo surgery with 25%.

**Conclusions:**

Postoperative coronary angiography is a useful tool with a significant therapeutic value. Pathological findings mandate further revascularization therapy in roughly half of the patients. PCI is a safe choice in the majority of patients, redo surgery is much less indicated.

## Background

Over the past decades and along with the numerous achievements in percutaneous coronary intervention, the patients referred for surgical revascularization progressively present with devastating coronary findings. The coronary artery bypass operation is the treatment of choice in advanced coronary artery disease, but cannot always guarantee complete revascularization in these patients with extreme calcifications and numerous stenotic lesions or coronary occlusions. As a consequence, the risk of perioperative myocardial infarction, which is reported to 2–8%, and the threat of bypass graft occlusion are increasing rather than decreasing nowadays. Immediately after surgery, a mild rise of troponin I and serum creatinine kinase (CK) may indicate such a prognostic relevant event, but does not actually prove it [1, 2]. Therefore, emergency coronary angiography can be performed to verify the diagnosis of an occluded coronary artery or bypass graft and to obtain a chance for immediate interventional therapy, and maybe also to reduce further costs for the health care system [3, 4].

The aim of the study was to evaluate the impact and benefit of control coronary angiography after coronary artery bypass surgery in case of suspected postoperative myocardial ischemia.

## Methods

### Patient cohort

All patients who demonstrated signs of myocardial ischemia during the postoperative course after isolated CABG surgery and underwent coronary angiography from 6/2008 to 06/2015 were analyzed in a retrospective study. Main inclusion criterion was myocardial ischemia post CABG defined as an increase of CK-MB beyond 100 U/l with a CK/CK-MB fraction exceeding > 10% within 48 h after uneventful surgery. If there were a steady increase or concomitant symptoms like malignant arrhythmias (ventricular tachycardia, ventricular fibrillation) or low output syndrome (cardiac index < 2.0 L/min/m^2^, worsening of pump function, or inadequate need of epinephrine), indication for coronary angiography was established earlier, when CK-MB levels had not surpassed the 100 U/l limit. Troponin levels were not analyzed because they were not available during the early years of the study, and because the analysis tool changed several times thereafter. Coronary angiography was performed regardless of serum creatinine levels. The only exclusion criterion for coronary angiography was a lack of therapeutic consequences, i.e. when no further PCI or surgical intervention was feasible.

### Coronary bypass surgery

The standard operative technique employed for coronary revascularization included the installation of cardiopulmonary bypass and cardioplegic arrest. Few patients underwent offpump surgery. After median sternotomy the left internal thoracic artery was harvested as a pedicled or skeletonized vessel according to the surgeon’s discretion. The right internal mammary artery and/or the left radial artery were prepared additionally in suitable patients younger than 70 years. Vein grafts were chosen mainly for the right coronary artery or in elderly patients. They were mostly prepared via small segmental skin incisions. For extracorporeal circulation either a standard heart-lung machine or minimized circuit was connected after cannulation of ascending aorta and right atrium in a standard fashion. Blood cardioplegia was routine regardless of extracorporeal circulation system. After completing coronary anastomosis meticulous transit time flow and pulsatility index measurements were obtained at all grafted vessels to test for patency and effectiveness.

### Postoperative management

Immediately after arrival at the intensive care unit (ICU) the patient was stabilized with appropriate fluid replacement and catecholamine administration. Serum probes were taken to analyze markers for myocardial ischemia including creatinine kinase (CK) and its CK-MB fraction. Blood chemistry was repeated every 6 h until referral to intermediate care or to the general ward. Continuous monitoring of the pressure lines and electrocardiogram (ECG) were supplemented by a 12-channel ECG recording after ICU admission. In case of significant CK-MB increase after surgery beyond 100 U/l or lacking apt CK-MB decrease, postoperative coronary angiography was asked for to rule out a graft problem.

### Statistical analysis

All data from the institutional database were retrospectively analyzed employing the SigmaPlot 11.0 software (Systat Software, Inc). The local ethics committee approved the study and waived individual informed consent. The various patient parameters were expressed as mean and standard deviation. Comparison between groups was achieved with the Wilcoxon U test and chi-square test where appropriate. *P* < 0.05 was considered significant in all tests.

## Results

### Incidence

During the 7 year study interval, 108 patients underwent postoperative coronary angiography after surgical coronary revascularization. There were 83 male and 25 female patients with a mean age of 66 ± 9 years. Considering the whole patient cohort (*n* = 4825) operated upon during that time, the incidence was calculated to 2.2%.

### Angiography findings

Twenty-nine patients showed intact bypass grafts with patent anastomosis and sufficient flow (27%). Seventy-nine out of the 108 patients (73%) demonstrated graft pathologies. Overall, 132 pathological findings were evident during repeat angiography averaging 1.7 per patient involved. The dominant problem was graft stenosis (64%), whereas anastomotic narrowing was less frequent (26%). Kinking of graft was noted in 10% of cases (Table 1).

**Table 1:** Angiography findings

Normal – intact grafts	29
Graft pathology	
Anastomostic stenosis	34
Graft stenosis arterial graft	31
venous graft	54
Graft kinking	13
Total	132 (1.7 per patient)

Seventeen patients (16%) had only minor lesions either not amenable to percutaneous treatment (PCI) or not considered meaningful to treat interventionally. There was a singular graft problem only in these cases. Thus, in a total of 46 patients (43%), treatment was not altered.

Accordingly, 62 patients were contemplated for additional treatment. Interestingly, the prevalence of anastomotic as well as arterial and vein graft stenosis, and kinking phenomena were not significantly different to the patient group without further coronary reintervention (Table 2).

**Table 2:** Spectrum of pathological findings

	Reintervention	No reintervention	Significance level
Stenosis of distal anastomosis	30 (48%)	4 (9%)	*p* = 0.095
Kinking of bypass graft	9 (15%)	4 (9%)	*p* = 0.419
Stenosis of arterial graft	25 (40%)	6 (13%)	*p* = 0.687
Stenosis of venous graft	39 (63%)	15 (33%)	*p* = 0.179
Total	103 (1.7 per patient)	29 (0.6 per patient)	

In patients after redo coronary revascularization, postoperative coronary angiography always demonstrated patent grafts. Patients with previous PTCA and/or stent placement showed comparable findings (6.9% vs. 5.9%, *p* > 0.05.) overall.

### Therapeutic consequences

Of the 62 patients being scheduled for additional treatment (57%), 10 patients (9%) underwent immediate redo surgery. A percutaneous intervention with stent placement was considered appropriate in 52 patients (48%) (Table 3).

**Table 3:** Therapeutic consequences related to pathology

	Reintervention	No reintervention	Total
Vessel pathology during angiography	Surgery 12 PCI 50	17	79
Normal findings	–	29	29
Total	62	46	108

*PCI* percutaneous coronary intervention

### Outcome

**Fig. 1:**
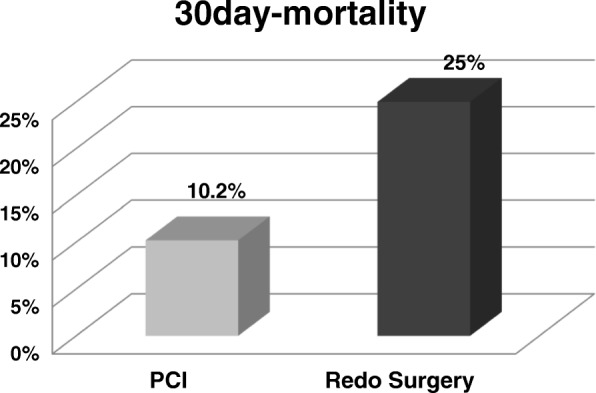
Outcome after reintervention

Patients whose angiograms revealed no need for reintervention had a 30d-mortality rate of 2.2%. When reintervention was needed, 30d-mortality rate rose to 13%. Mortality following PCI was 10.2%, and much more pronounced after redo surgery with 25% (Fig. 1).

### Risk factors

When comparing patients with and without the need for reintervention, demographic data including the extent of the coronary artery disease, left main stenosis, risk factors for coronary artery disease, left ventricular ejection fraction, comorbidities, NYHA level, EuroScore, and acuity of operation revealed no significant differences except for a higher proportion of obese patients in the reintervention group (Table 4) When comparing patients undergoing PCI versus surgical revision following coronary angiography, previous PTCA and/or stent placement was more often noted in the redo surgery subgroup (33% vs. 8%, *p* = 0.019).

**Table 4:** Demographics related to the necessity of PCI and surgery

	PCI	Surgery	Reintervention	No reintervention	Significance level^b^
Age (years)	66.8 ± 9	65.8 ± 6	66.6 ± 8	65.7 ± 10	*p* = 0.321
Gender (m/f)	37/15	9/1	46/16	37/9	*p* = 0.447
CAD – 1VD	1	0	1 (1.6%)	3 (6.5%)	
2VD	3	1	4 (6.5%)	4 (8.6%)	*p* = 0.394
3VD	46	11	57 (91.9%)	41 (81.9%)	
Left main stem stenosis	20	9	29 (46.7%)	23 (50.0%)	*p* = 0.740
Acute myocardial infarction	12	3	15 (24.1%)	8 (17.3%)	*p* = 0.729
s/p CABG	2	1	3 (4.8%)	3 (6.5%)	*p* = 0.142
Hypertension	41	10	51 (82.2%)	34 (73.9%)	*p* = 0.294
Hyperlipidemia	38	10	48 (77.4%)	31 (67.3%)	*p* = 0.244
Nicotine abuse	19	2	21 (33.8%)	19 (34.7%)	*p* = 0.625
Diabetes mellitus	13	2	15 (24.1%)	15 (32.6%)	*p* = 0.932
Obesity (BMI > 30)	7	2	9 (14.5%)	15 (32.6%)	*p* = 0.025
Peripheral vascular disease	10	2	12 (19.3%)	10 (21.7%)	*p* = 0.092
Renal disease	2	0	2 (3.2%)	2 (4.3%)	*p* = 0.760
Pulmonary disease	4	0	4 (6.5%)	8 (17.3%)	*p* = 0.073
Cerebral disease	4	1	5 (8.1%)	4 (8.6%)	*p* = 0.906
Cardiac arrhythmias	1	0	1 (1.6%)	2 (4.3%)	*p* = 0.392
LV ejection fraction	58 ± 15%	56 ± 12%	58 ± 14%	59 ± 11%	*p* = 0.732
NYHA I	11	5	16 (25.8%)	4 (8.6%)	
NYHA II	11	1	12 (19.3%)	10 (21.7%)	*p* = 0.148
NYHA III	26	5	31 (50.0%)	30 (65.2%)	
NYHA IV	2	1	3 (4.8%)	2 (8.6%)	
EuroScore 0–2	13	5	18 (36.0%)^a^	12 (35.3%)^a^	
EuroScore 3–5	20	4	24 (48.0%)^a^	10 (29.4%)^a^	*p* = 0.086^a^
EuroScore > 5	6	2	8 (16.0%)^a^	12 (35.3%)^a^	
Elective surgery	25	4	29 (46.7%)	23 (50.0%)	
Urgent surgery	21	7	28 (45.2%)	17 (36.9%)	*p* = 0.569
Emergency surgery	4	1	5 (8.1%)	6 (13.1%)	

*PCI* percutaneous coronary intervention, *CAD* coronary artery disease, *CABG* coronary artery bypass operation, ^a^ = incomplete data, ^b^ Comparison of reintervention versus no reintervention

The surgical technique, i.e. using extracorporeal circulation, minimized extracorporeal circulation, or operating offpump, did not have an impact too. Reintervention rates were comparable. Likewise, the utilization of arterial and venous grafts was similar as were the coronary targets. The indication for postoperative angiography was mainly based on the increase of serum CK/CK-MB levels in both the reintervention (90.3%) and the no-reintervention group (89.1%), whereas low cardiac output, malignant arrhythmias and the necessity of resuscitation were much less contributing. There were no significant differences except for the higher incidence of low cardiac output in the intervention group (Table 5).

**Table 5:** Perioperative parameters related to the necessity of PCI and surgery

	PCI	Surgery	Reintervention	No reintervention	Significance level^a^
Surgical technique:					
ECC	30	7	37 (59.7%)	31 (67.4%)	*p* = 0.673
MECC	18	5	23 (37.1%)	11 (23.9%)	*p* = 0.209
OPCAB	2	0	2 (3.2%)	4 (8.7%)	*p* = 0.219
Grafts:					
LITA	45	11	56 (90.3%)	39 (82.1%)	*p* = 0.979
RITA	2	1	3 (4.8%)	4 (8.7%)	*p* = 0.381
Radial artery	4	1	5 (8.1%)	1 (2.2%)	*p* = 0.216
Venous graft	47	12	59 (95.2%)	42 (91.3%)	*p* = 0.901
Coronary anastomosis:					
LAD	49 (98.0%)	11 (91.7%)	59 (95.2%)	44 (95.7%)	*p* = 0.905
RCX	31 (62.0%)	5 (41.7%)	36 (58.1%)	26 (56.5%)	*p* = 0.642
RCA	34 (68.0%)	8 (66.7%)	42 (67.7%)	28 (60.9%)	*p* = 0.459
Indication for postop. Angiography					
Increase CK/CK-MB levels	46	10	56 (90.3%)	41 (89.1%)	*p* = 0.612
LCO	13	3	16 (25.8%)	4 (8.7%)	*p* = 0.040
Arrhythmias (new onset, maligne)	7	0	7 (11.3%)	9 (19.6%)	*p* = 0.183
Resuscitation for cardiac arrest	5	0	5 (8.1%)	4 (8.7%)	*p* = 0.820

*PCI* percutaneous coronary intervention, *ECC* standard extracorporeal circulation, *MECC* minimized extracorporeal circulation, *OPCAB* offpump coronary artery bypass surgery, *LITA* left internal mammary artery, *RITA* right internal mammary artery, *CK* serum creatinine kinase, *LCO* low output syndrome

^a^Comprison of reintervention versus no reintervention

A more detailed analysis of postoperative serum CK/CK-MB levels was not predictive too. Neither serum CK, nor serum CK-MB, nor the CK/CK-MB ratio was significantly different between the reinvention group and no-reintervention group.

## Discussion

Coronary bypass surgery is fundamental in the treatment of coronary artery disease. Its indication is mainly based on findings during coronary angiography and well defined by national and international guidelines [5, 6]. On contrast, the diagnosis of post−/perioperative myocardial ischemia is grounded on elevated serum creatinine kinase (CK), CK-MB or cardiac troponin I (cTNI) levels, and the elevation of these serum markers within the first 24 h are associated with increased intermediate- and long-term mortality [1, 2]. However, there are neither uniform recommendations with regard what to enzymes to analyze nor cut-off levels indicating a need for further diagnostic or therapeutic interventions. Likewise, discussion is controversial whether cTNI or CK-MB is more useful to identify perioperative myocardial infarction [7, 8]. Yet, repeat coronary angiography remains the gold standard to evaluate postoperative myocardial ischemia, its indication has been recently proposed in a algorithm of the ESC Joint Working Groups on Cardiovascular Surgery and the Cellular Biology of the Heart position paper [3]. It allows to judge patency of bypass grafts or the presence of untreated native coronary vessels as there is a substantial overlap with patients without graft occlusion, meaning that the patency status in the individual cannot be reliably predicted from these noninvasive tests [9]. Nevertheless, the usefulness of repeat angiography is still under debate as it not only augments costs of treatment and may prolong hospital stay, especially if no further consequences are drawn, but also exposes the patient to an additional risk. According to the German Society of Cardiology the mortality for coronary angiography is 0.4–0.7% if no coronary artery disease is present, and 3.7% in patients with acute coronary syndrome (Press release German Society of Cardiology 10/2014).

The incidence of postoperative angiography after CABG in the literature is ranging from 0.4 to 30% depending on the cut-off levels within the institutional protocol [10–16]. Findings were reported to be normal in 42 to 67% of patients. Graft pathologies including graft occlusion, stenosis, incorrect anastomosis, incomplete revascularization or poor distal run-off were noted in 33 to 58% of cases [10–13]. In our institution, we had an incidence for repeat angiography of 2.2% only, graft pathologies were evident in 73% of patients. It may well be that in some few cases coronary angiography was deemed unnecessary due to individual decisions. However, the overall number has not been evaluated. The assumed more than average prevalence of pathologic findings might be a consequence of our more than average nation-wide patient sickness. We did not scrutinize native coronary vessels in redo angiograms, since the extent of coronary vessel revascularization is often ambiguously discussed among colleagues.

Not all patients with conspicuous angiography findings following coronary artery bypass surgery underwent further therapeutic interventions. In the literature, the cohort of patients treated medically only comprises about 10 to 20% of patients. We had 16% of cases being conservatively treated without another revascularization procedure [10–14]. Thus, in a total of 46 patients (42.5%) repeat angiography did not alter the current treatment plan.

If angiography findings advocate subsequent coronary treatment, the investigating cardiologist should immediately contact the respective cardiac surgeon to discuss the further options [17]. A percutaneous intervention with balloon dilatation and stent placement is mostly favored for its technical ease and patient’s comfort. Patent grafts may lower the risk myocardial ischemia during PCI. However, interventional therapy is not always feasible, especially in totally occluded vessels or grafts. Moreover, fragile anastomoses and endarterectomies are frequently not amenable for PCI. Unless totally thrombosed, native coronary vessels and grafts can be approached again by surgical means, i.e. redo CABG surgery. Nevertheless, many surgeons are reluctant with regard to repeat surgery for its unfavorable prognosis. Accordingly, its incidence is usually well below 20%. In our institution, only 11% of patients went back to OR as the surgical risk was mostly considered to be higher for redo surgery as compared to PCI. Surgery was mainly indicated in case of significant distal anastomotic graft stenosis at a proximally occluded coronary vessel. It is noteworthy that we found these patients to have a higher incidence of prior PCI treatment. Thus, patient with prior PCI seem to have a higher risk for redo CABG in case of pathologic angiography.

Mortality is strongly influenced by perioperative myocardial ischemia. Some groups reported significant differences in CK and troponin levels between the groups, while others did not [13, 14]. Several reports well prove that a postoperative increase of cTnI or CK-MB is associated with a worse prognosis [1, 2]. Accordingly, pathologic angiography may certainly alter outcome. Our overall mortality rate rose from 2.2 to 13% with the necessity for postoperative interventions, and was elevated up to 25% after redo surgery. Comparable findings have been reported by Thielmann et al. with 12% in-hospital mortality after PCI, 20% following redo surgery, and 14.8% with medical treatment [13]. Since this dramatic increase of mortality in urgent redo surgery has also been reported by others, surgeons probably will remain reluctant referring a patient back to the operation room [10]. Obviously, the second surgical procedure exposes the patient to considerable operative trauma which cannot be easily overcome. Considering our more and more elderly and comorbid patient population for coronary artery bypass surgery PCI will be favored to limit the extent of myocardial damage whenever possible.

Prediction of necessity and usefulness of repeat angiography is hardly possible. In about 90% of patients indication for angiography was based on an increase of serum CK/CK-MB markers in our institution. However, there was no difference between the reintervention group and the no-intervention group. Likewise, malignant arrhythmias and resuscitation failed as predictor. In our patient cohort, only the incidence of low cardiac output with shock was significantly higher in the intervention group. The best way to deal with the problem, i.e. to the minimize the need for postoperative interventions, would offer an intraoperative angiogram.

### Study limitations

The manuscript is mainly limited by the following. [1] As only symptomatic patients underwent postoperative coronary catheterization by the cardiology team, the incidence of silent graft occlusion cannot be estimated from the study. [2] The focus of the retrospective analysis was graft complications only, i.e. untouched native coronary vessel occlusion was not investigated. The idea behind the latter was to reduce a bias since some surgeons perform CABG more aggressively than others who try to avoid uncertain graft anastomoses. For most surgeons and physicians an occluded graft anastomosis is seen as surgical mistake, whereas an occluded native vessel is considered fate.

## Conclusion

The percentage of CABG patients undergoing repeat angiography is low. A liberal indication for angiography seems justified as pathological findings mandate further revascularization therapy in roughly half of the patients. PCI is a safe choice in the majority of patients, redo surgery is much less indicated.

## Abbreviations

BMI: Body mass index
CABG: Coronary bypass graft
CAD: Coronary artery disease
CK: Serum creatinine kinase
CK-MB: Serum creatinine kinase muscle brain
cTNI: Cardiac troponin I
ECC: Standard extracorporeal circulation
ECG: Electrocardiogram
ICU: Intensive care unit
LAD: Left anterior descending artery
LCA: Left circumflex artery
LCO: Low cardiac output
LITA: Left internal mammary artery
MECC: Minimized extracorporeal circulation
NYHA: New York Heart Association
OPCAB: Off-pump coronary artery bypass surgery
PCI: Percutaneous coronary intervention
PTCA: Percutaneous transluminal coronary angioplasty
RCA: Right coronary artery
RITA: Right internal mammary artery
